# Physical examination in undergraduate medical education in the field of general practice – a scoping review

**DOI:** 10.1186/s12909-017-1074-1

**Published:** 2017-11-25

**Authors:** Dirk Moßhammer, Joachim Graf, Stefanie Joos, Rebekka Hertkorn

**Affiliations:** 1 University Hospital Tuebingen, Institute for General Medicine and Interprofessional Care, Österbergstraße 9, D-72074 Tuebingen, Germany; 2 Department of Women’s Health, Research Institute for Women’s Health, University Hospital Tuebingen, Calwerstraße 7, D-72076 Tuebingen, Germany; 3 Medical Faculty Tuebingen, Dean’s Office for Students’ Affairs, Geissweg 5, D-72076 Tuebingen, Germany

**Keywords:** Physical examination, Medical education, Medical students, Teaching methods, Scoping review

## Abstract

**Background:**

Physical examination (PE) is an essential clinical skill and a central part of a physician’s daily activity. Teaching of PE has been integrated into medical school by many clinical disciplines with respective specific examination procedures. For instance, PE teaching in general practice may include a full-body examination approach. Studies show that PE-skills of medical students often need enhancement. The aim of this article was to scope the literature regarding the teaching and research of PE within general practice during undergraduate medical education. We evaluated a wide breadth of literature relating to the content, study design, country of research institution and year of publication.

**Methods:**

Literature search in Medline along the PRISMA-P protocol was performed by search syntax (“physical examination” AND “medical education” AND “undergraduate” AND general practice) considering Medline MeSH (Medical Subject Heading)-Terms and Medline search term tree structure. Independent title, abstract and full-text screening with defined inclusion and exclusion criteria was performed. Full texts were analyzed by publication year, country of origin, study design and content (by categorizing articles along their main topic according to qualitative content analysis of Mayring).

**Results:**

One-hundred seven articles were included. The annual number of publications ranged from 4 to 14 and had a slightly rising trend since 2000. Nearly half of the publications originated from the United States (*n* = 54), 33 from Canada and the United Kingdom. Overall, intervention studies represented the largest group (*n* = 60, including uncontrolled and controlled studies, randomized and non-randomized), followed by cross-sectional studies (*n* = 29). The 117 studies could be assigned to five categories “teaching methods (*n* = 53)”, “teaching quality (*n* = 33)”, “performance evaluation and examination formats (n=19)”, “students’ views (*n* = 8)” and “patients’ and standardized patients’ views (n=4)”.

**Conclusions:**

The present work shows a wide spectrum of teaching and research activities and a certain level of evidence for the effectiveness of individual teaching methods. It can be used as orientation and impulse generator for the further development of medical education in the field of PE.

## Background

### Physical examination in undergraduate medical education: Overview

Physical examination (PE) is an essential clinical skill and a central part of a physician’s daily activity [1]. PE examination and communication skills are of crucial importance for the doctor-patient relationship, patient safety and the efficiency of medical treatment [2–4]. It is known that the risk of medical errors and adverse events can be significantly reduced by adequate PE [5]. Further, it can be assumed that inappropriate use or overuse of diagnostic technology can be reduced by means of preceding evidence-based PE [6]. Recent studies indicate that even final year students still lack in PE skills [7]. Teaching of PE has been integrated into medical school by many clinical disciplines with respective specific examination procedures. For instance, PE teaching in general practice may include a full-body examination approach. Internationally, there is broad discussion about the appropriateness of teaching methods and concepts, and the requirement for teaching of specific PE techniques [8–11]. Also, the learning environment needs to be considered (e.g. learning/teaching in hospital or in practices) [12]. Overall, there is no evidence favoring one approach. With this background, the Institute of General Practice and Interprofessional Care of the University of Tuebingen has been developing a PE course that focuses on teaching a head-to-toe examination (HTTE) in small groups with their third-year medical students and standardized patients. This HTTE suggests a scheme that focuses on minimising re-positioning of the person examined and considers core examination steps suggested by Gowda et al. [10, 13]. The evaluation of this course is embedded at the end of each semester in an OSCE (Objective Structured Clinical Examination)-structure [14–16]. Development and implementation of this course [13] was further to a scope of the literature of PE [17].

### Aims and objectives

The aim of this article was to scope the literature for teaching and research activities in the field of PE in undergraduate medical education. The scope review was performed to answer the following research questions:

How broad is the range of literature regarding different methods of teaching PE competences and which dimensions of assessment can be distinguished?

Several sub-questions can be derived from the research question: Which factors determine the quality of physical examination teaching in undergraduate medical education?

How can the identified studies be analyzed regarding study design, country of research institution and year of publication? How can the studies be assessed in qualitative terms, how many categories can the results be concretely assigned to, how many and which teaching methods are differentiated?

## Methods

### General characteristics

Evidence maps and scoping reviews are innovative approaches of systematic evidence processing for generating an overview of the literature about a specific topic, usually presented in tabular form or as a web-based database or narrative description. Scoping reviews often act as precursors systemic review by doing a narrative integration of the relevant evidence. In contrast to systematic reviews, scoping reviews are characterized by the following [18]:

- Research question is more broadly defined than in systematic reviews in relation to regarding outcomes, study design, comparison groups as well as inclusion and exclusion criteria. While at systemtic reviews the literature is determined on the basis of clearly defined research questions, research questions in scoping reviews are broadened to include several types of studies and to identify research gaps. The identification of research gapsis particularly important for interventions which are used without sufficient evidence. In contrast, systematic reviews are mainly used to estimate effects for interventions and evaluate whether the included studies are reliable.
- Inclusion and exclusion criteria are rarely defined a priori, but are developed, modified or extended during the work process
- Quality assessment of the included studies is usually not performed (unless there are already systematic reviews of the topic range). Detailed analysis/assessment of the interventions, control groups or outcome measures is not the focus of evidence mapping or scoping reviews

Presentation of results is mostly tabular or narrative [18–22]. The literature analysis of the current manuscript was made in preparation of the HTTE course in Tuebingen. We conducted a scoping review with the aim of mapping the existing literature in a field of interest in terms of the volume, nature, and characteristics of the primary research. The approach we used was based on the methodology of Schmucker et al. [18], Peters et al. [20] and Levac et al. [21]. The focus of this scoping review is teaching and research activities in undergraduate medical education in the field of PE. We focused on quantitative and qualitative dimensions of contemplable studies, but the evidence of individual PE procedures was not assessed.

### Database and search syntax

According to PRISMA a systematic literature search in PubMed was performed in March 2015 [22]. The search syntax (“physical examination” AND “medical education” AND “undergraduate”) considered the Medline MeSH (Medical Subject Heading)-Terms and the Medline search term tree structure.

### Literature screening, exclusion and inclusion criteria

Before a systematic literature review was performed, inclusion and exclusion criteria were defined: We included all articles published in the last 15 years, which could be found by the use of the described search syntax and with a focus on general practice. Exclusion criteria were language (language other than English and German), age, low evidence and focus on other disciplines than general practice. Articles in languages other than English or German were excluded due to languages barriers. Comments, editorials or letters to the editor, as well as articles, which were published before the year 2000, were not included because of limited access to abstracts and full texts as well as restrictions in feasibility. We considered articles concerning undergraduate medical education with the date of publication between January 2000 and March 2015 (qualitative and quantitative studies with and without control group). Studies in the field of emergency medicine, obstetrics, gynecology, dentistry, pediatrics, ophthalmology were excluded. We decided to exclude studies referring to these fields because the focus was physical examination by general practitioners (I.E. relevant examination techniques for general physical examination in medical practice). Of course emergency medicine is also a part of general medicine but we felt that it is less relevant for the teaching in this area because other specialties are more involved.

**Fig. 1:**
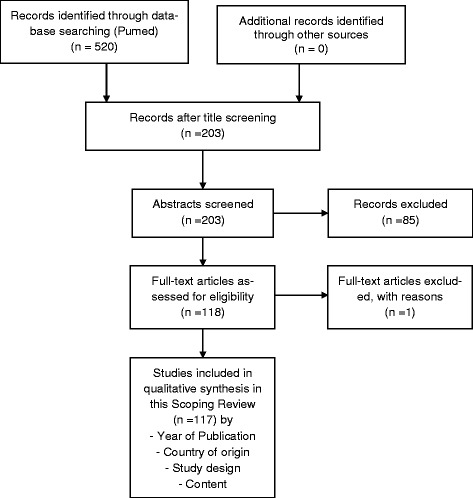
Overview of literature management

Following the application of the aforementioned criteria we identified *n* = 520 titles. All identified titles of the search results were screened as per the PRISMA diagram, whereby all titles without relevance for the research question (*n* = 317) were eliminated. After that the remaining *n* = 203 abstracts were screened, which resulted in the exclusion of *n* = 85 further records. Hereupon, *n* = 118 relevant articles were read. One paper was excluded because the content was not relevant to the research question.Therefore the total number of included articles amounted to *n* = 117. So, it can be specified that (in accordance with the PRISMA diagram and the principles of scoping reviews) literature screening was refined at each step of the search process: A consensus procedure for proposed inclusion or exclusion of studies was performed at each step. Furthermore we concentrated on examination techniques relevant to general physical examination in medical practice because of focus is on the general practice context. Figure 1 presents an overview of the literature review as a PRISMA diagram.

Titles, abstracts and full texts were screened independently by the two authors D.M. and R.H. in accordance with the quality assessment methodology of scoping reviews (Fig. 1).

### Analysis and presentation

For the planning, conduction and results presentation of this Scoping Review, established and international methodology literature were considered [19, 20, 22]. *N* = 117 full texts were analyzed by publication year, country of origin, study design and content. Supplementary material (see under results) was developed along the PICOS-scheme (Patients - medical students in this instance -; **I**ntervention; **C**ontrol [control group/comparison]; **O**utcome; **S**tudy Design) [23].

#### Classification of study designs

**Fig. 2:**
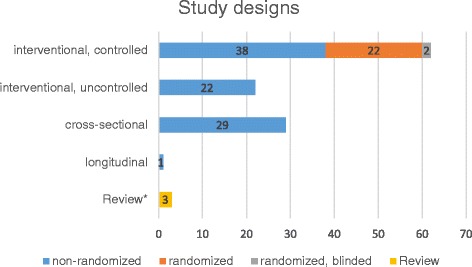
Distribution of study designs

Intervention studies are studies with two or more interventions (e. g. comparison of learning materials or teaching staff) or studies in which one intervention was compared with an existing standard (see Fig. 2).

#### Classification/categorization of content

With regard to content, all articles were assigned to categories/subcategories (see Table 1). These categories/subcategories were developed based on the content of the articles in an iterative process conducted by the authors according to The Qualitative Content Analysis of Mayring methodology (I.E. by generating *deductive categories/subcategories* (along explicitly given information in the articles) and *inductive categories/subcategories* (along implicitly given information)) [24].

The main idea of the *deductive* approach is to give explicit definitions, coding rules and examples for each deductive category, determining exactly under what circumstances a passage of text can be coded with a category. The category definitions are put together within a coding agenda. The qualitative analysis consists of a methodological controlled 6-step assignment of the passage of text to a category. The main idea of the *inductive* approach is to formulate a criterion for definition, derived from the background theory and the research question itself; this determines which text is considered. With the criterion established, the material is worked through and categories are tentatively established and adjusted using another six-step model [24]. The aim is to deduce consistent, demarcated, valid categories, in which all codes could be appropriately applied.

Since the categories were derived from the text material and were not prepared before the material was screened, the *inductive approach* was applied in the present case. Initially, the central questionnaire, and inclusion/ exclusion criteria were defined (step 1). After this, the category definitions were defined (step 2) and the individual categories were gradually derived inductively from the screened texts (step 3). The categories derived from these were initially provisional, and were further revised after screening of 50% of all texts, (step 4), which also resulted in a revision of the category definitions and the central research question (formal reliability test). Afterwards, all missing texts were screened and the categories that had been derived so far were expanded (step 5), in order to finally define the categories and to assign to respective categories after a new review of all texts (summative reliability test). Subcategories were also derived. Finally, it was quantified how often the individual categories were represented in the text material (step 6).

The Content Analyses of Mayring was performed independently from each other by the two authors D.M. and R.H. After a first screening of all *n* = 117 articles, both authors defined the central five categories in the context of summative reliability testing (i.e. after step 5), whereupon both authors independently matched the articles to the categories again independently from each other. Thereby, every article represents one thematic code and matches one category without any overlapping. Inter-rater reliability amounted to 0.88 (*n* = 103), because 14 articles were dedicated differently. These disagreements were eliminated by discussing in a communicative validation process including all 4 authors (D.M., R.H., J.G., S.J.). If there was more than one thematic focus in the text (e.g. if an article also evaluated the student view of a specific teaching method), we categorized it according to the main focus of the article.

For clarity, categories are exemplified by a set of articles relating to that category. Full information on articles of a certain category can be found in the supplement material (available on request, see under point 6: Declarations, availability of data and materials).

## Results

The following analyses concern publication year, country of origin, study design and content of the included 117 articles.

### Year of publication and countries of origin

The annual number of publications ranged from 4 to 14 and had a slightly rising trend since 2000. The most papers (*n* = 14) were published in the year 2009, followed by the years 2014 (*n* = 13) and 2004 (*n* = 11).

Nearly half of the publications originate from the United States (*n* = 54). *N* = 17 came from Canada, followed by the United Kingdom (*n* = 16). Only a few publications came from countries in Europe (Europe without UK: *n* = 10), the Middle East, and Asia (*n* = 9), Australia (*n* = 7), New Zealand (*n* = 2), South America (Brasil, *n* = 1), Africa (South Africa, *n* = 1). The contributions from the European countries were as follows: Netherlands with four publications, Germany and Ireland with two publications each, Norway and Switzerland with one publication each.

### Study designs

Overall, intervention studies represented the largest group (*n* = 60, including intervention studies with and without control groups), followed by cross-sectional studies without control group (*n* = 29). One follow-up (longitudinal) study was found, and among the three review articles, there were one systematic review and two unsystematic reviews (see Fig. 2).

### Content

During the analysis process the identified articles were matched into the following 5 categories (CAT): “teaching methods” (CAT I), “teaching quality” (CAT II), “performance evaluation and assessment formats” (CAT III), “student views” (CAT IV) and “patient/ standardized patient perspective” (CAT V). All categories consist of different subcategories (CAT I: 4; CAT II: 3; CAT III: 3; CAT IV: 4; CAT V: 1) (Table 1).

**Table 1:** With regard to content categorisation of the 117 articles on physical examination in undergraduate medical education

Category No.	Category	Subcategory	Number of fulltexts
CAT I	Teaching methods^a^ (*n* = 53)	computer−/internet-based	11
		Learning materials (pocket cards, checklists)	2
		Concepts (GALS, hypothesis-driven or problem-based learning, mentoring programs)	4
		Practical courses	
		⋅ simulation-based learning	11
		⋅ peer-assisted learning	6
		⋅ bedside teaching	3
		⋅ OSCE	2
		⋅ Anatomy courses	2
		⋅ ultrasound-controlled (percussion of the Adomen, e. g. liver size estimation)	1
		⋅ others	11
CAT II	Teaching quality (*n* = 33)	Qualification of the teachers	16
		content quality	15
		learning environment	2
CAT III	Performance evaluation and examination formats (*n* = 19)	quality of evaluation instrument (reliability/validity)	16
		development/introduction of assessment tools	2
		correlation with exam results	1
CAT IV	Students‘view (*n* = 8)	peer physical examination	5
		standardized patients and real patients	1
		OSCE as assessment format	1
		digital rectal examination	1
CAT V	Patients‘and standardized patients‘view (*n* = 4)	in terms of their participation in medical education	4

^a^including teaching material and learning concepts, see text

*GALS* Gait-Arm-Legs-and-Spine-locomotor-screening

*OSCE* Objective Structured Clinical Examination

### Teaching methods

This category represented the largest group with a total of *n* = 53 studies. The four subcategories “computer/internet based”, “learning materials”, “teaching concepts” and “practical courses” were assigned to this category. The largest subcategory “practical courses” was further divided.

#### Computer/internet based

Eight articles investigated the benefit of computer and internet based learning programs to acquire clinical examination skills [25–32]. These additional learning programs were rated quite positively by students [31, 32], or students were scored higher in exams (knowledge based and skill based assessment, p.e. OSCE) after using computer based learning programs compared to students without IT support [25, 28–30]. Two controlled intervention studies on the additional value of the use of CDs for learning cardiac auscultation showed improved detection rates of heart murmurs in simulated environments in the intervention group [33, 34].

#### Learning material

In two uncontrolled intervention studies the use of pocket cards and checklists as supplementary learning material were analyzed [35, 36]. In the study by Torre et al. the use of pocket cards was positively rated by the majority of students [35]. Altschuler et al. found that students achieved better results in a skill based assessment of an investigator, whereby the investigator acted as the standardized patient and the evaluator. The results after a two-week physical medicine clerkship using checklists in 19 of 20 of musculoskeletal examination maneuvers was compared to the results acquired before the clerkship [36].

#### Concepts

The didactic approaches of problem-based learning and hypothesis-driven learning were the focus of two studies: In the study of Chen et al. students encountered standardized patients with defined complaints in a clinical setting. Then a symptom-related clinical examination had to be performed. Students favored the problem-based learning approaches including simulation patients for learning PE skills [37]. In the study of Yudkowsky et al. students were given clinical cases. Before PE of the patient, students listed anticipated positive PE findings for plausible diagnosis. The students performed 88% of the maneuvers correctly [38].

### Practical courses

#### Simulation based learning

Studies regarding the use of electronic/digital stethoscopes are the largest group from this subcategory. Three studies were found regarding this topic [39–41]: Two randomized controlled studies concluded different results in the detection of heart sounds and heart murmurs, depending on the use of electronic stethoscopes. Høyte et al. found no differences in the detection rates depending on the stethoscope usage [41]. Better detection rates were found by Mesquita et al. in the auscultation of heart sounds generated by a software [39].

In three controlled intervention trials, the additional performance of simulated heart sounds led to better detection rates [42–44]. Swamy et al. compared practicing chest examination on a simulator with practicing on each other and found no significant differences in chest examination skills between the two groups. However significant improvement of students’ knowledge was found in the group of students who obtained their practical skills on the simulator [45]. Siebeck et al. compared rectal examination on the simulator and on standardized patients and found no differences in the increase of knowledge. However, compared to students in the simulator group, students in the standardized patient group reported a significant reduction in terms of overcoming their inhibition [46].

#### Peer-assisted learning

In this subgroup four studies investigated the impact of learning results in peer groups. In three studies, an increase of practical skills could be shown. Overall, the feedback from the teachers and students was positive [47–50].

#### Bedside teaching

In this subcategory three studies were assigned. Smith et al. compared examination outcomes (OSCE hand and knee examination), student evaluation and satisfaction for structured clinical instruction modules (SCIM) to small group bedside tutorials [51]. No difference in the hand OSCE was found but there was a better outcome in knee examination in the bedside teaching group. There were no differences in students’ satisfaction. Roberts et al. assessed the success of twice-weekly bedside diagnosis rounds for 3rd-year medical students during their medicine clerkship [52]. Compared with students of the ‘traditional’ system who did not undergo the bedside teaching, the students who were trained had an overall higher OSCE physical exam score.

Kianmehr et al. evaluated in their cross-sectional study the perspective of medical students and patients on bedside teaching [53]. Most of the medical students believed that bedside teaching is effective for learning alongside other physical examination skills and 40% thought that bedside teaching is the most effective way of learning clinical skills. Sixty percent of patients were comfortable with bedside teaching.

#### Others

Examples of studies assigned to this sub-category included one in which cadavers were used for practicing knee examination [54] and additional courses such as interdisciplinary workshops on PE [55]. A systematic review of effective teaching methods for musculoskeletal clinical examination skills included 24 studies. Most of the 18 studies related to undergraduate medical education and interactive small group teaching was favored [56].

### Teaching quality

This category represented the second largest group. *N* = 33 studies were assigned to the three subcategories “qualification of the teachers”, “content quality” and “teaching environment”.

#### Qualification of the teachers

Ten articles investigated the influence of teachers on the quality of teaching physical examination skills. Comparative studies predominated [57–66]. For instance the quality of teaching of PE by doctors was compared to that of nurses or more advanced students. Most studies showed no significant differences. In a randomized comparative study, Zeng et al. studied the difference between full-time faculty and part-time faculty. Students who were taught by full-time faculty showed better performance in PE skills [66].

#### Content quality

Coady et al. determined a core content of musculoskeletal examination on the basis of a survey among specialists. A total of 50 maneuvers were deemed core and to be taught to all medical students [67]. Woolf et al. developed core recommendations for a musculoskeletal undergraduate curriculum on the basis of an international survey [68]. Moore et al. determined 22 essential procedures of a neurological examination on the basis of a survey among neurologists [69]. Gowda et al. surveyed physical diagnosis course directors (PDCDs) and internal medicine clerkship directors (IMCDs) in the US and developed 37 maneuvers of a core physical examination [70].

#### Learning environment

Two studies were assigned to this subcategory. Barclay et al. compared the effect of the training location (hospital-based setting versus community setting) and found no significant differences of skills assessed in the OSCE (history taking and PE, interpersonal skills, patients’ satisfaction) [71]. Barnette et al. assessed students’ perceptions regarding same-gender versus mixed-gender partnering in practicing PE (excluding genital-rectal, breast and pelvic examination). Male students tended to feel more comfortable in the mixed-gender groups in all settings. Female students felt intimidated in mixed-gender groups, especially when they were in the role of patient with a male examiner [72].

### Performance evaluation and assessment formats

This category was the third largest group with *n* = 19 articles. It was divided into three subcategories, which are illustrated with examples.

#### Quality of the evaluation instrument (reliability, validity)

In this subcategory studies of the validity of checklists and worksheets and of the reliability and validity of examination formats (e. g. “Direct Observation Clinical Encounter Examination” [DOCEE]) are summarized. DOCEE is a practical examination format, which evaluates clinical skills with real patients in clinical settings [73–75]. Furthermore, studies investigating the effect of external factors on the performance of PE were assigned to this subcategory. For example, Doig et al. found that the reliability of the OSCE exam results were not significantly associated with the clinical background of examiners (senior medicine residents, family physicians, other specialists), the organization of the OSCE station and the time of the exam (morning versus afternoon session) [76].

#### Development/introduction of assessment tools

As an example, one study assigned to this subcategory investigated the use of “Objective Structured Video Examination” (OSVE). For this purpose, students were shown videos and were then asked questions in a written form regarding the content of the video clip. Nearly 70% of the tasks were solved correctly. OSVE did not contain a practical skills examination [77].

#### Correlation with exam results

Townsend et al. showed a positive correlation of OSCE results of students after a clinical attachment in general practice and the test results of their final medical school exams [78].

### Students‘views

Four subcategories with eight articles were assigned to this category [79–83].

#### Peer physical examination

Most articles addressing this topic were surveys [79–83]. One review of the literature was found [79–83]. The review covered a total of 23 studies, including the following: attitudes after participating in peer physical examination (PPE), fears and concerns of students regarding PPE, gender differences, body regions examined, influences by religion, ethnicity and cultural beliefs and strategies to improve the willingness to participate in PPE. In total, students showed a high willingness to participate with the exception of PPE of intimate body regions [79]. White students and non-religious students showed greater willingness to participate in PPE [81].

#### Standardized patients and real patients

Bokken et al. studied students’ perspectives in terms of strengths and weaknesses of real patients and standardized patients. Students found that the examination of real patients were more authentic and instructive, and that standardized patients would be suitable for preparation for patient contact and for practice of intimate examinations. In addition, students considered the feedback from standardized patients in terms of communication skills as positive [84].

#### OSCE as assessment format

Khursheed et al. conducted a survey regarding the topic Objective Structured Clinical Examination (OSCE). According to the students, OSCE was a practicable and helpful assessment format in terms of evaluating practical skills (in particular of a head-to-toe PE) [85].

#### Digital rectal examination

Lawrentschuk et al. asked students about digital rectal examination (DRE). 97% of the surveyed students found that DRE is an essential medical skill according to 94% of the respondents found that DRE should be trained by students until graduation. 92% reported to have been informed about DRE and on average students performed two DRE during medical school. 17% reported that they had never performed a DRE. The main reason for not performing DRE was the lack of available doctors to oversee the examination. About half of the respondents reported to be able to give a safe judgement based on their findings in the DRE [86].

### Patients’and standardized patients’ views

Four articles were assigned to this category. All of them were surveys among real patients (*n* = 3) and standardized patients (*n* = 1) concerning reasons for participation, and attitudes and experiences of patients as participants in PE [87–90].

Gandhi et al. explored the reasons of patients taking part in final year exams. Willingness to help was the most reported reason for participation. Only a few patients reported personal reasons for participation, for example to get an earlier operation or better care. Almost 74% had the feeling of having rendered a positive contribution to medical education. Nearly half of them liked to know how often the examination was performed in one session, and more than half of those patients liked to know whether other persons would also assist (for example peer students or tutors) [88]. Abe et al. surveyed standardized patients from Japan. 80% of the participants were willing to take part in the examination of the head, arms and legs. 25% were willing to take part in the examination of the thorax, back and abdomen. Male persons and persons over 50 years of age were more likely willing to take part in PE than female and younger persons [87].

## Discussion

### Relevance and discussion of the results

There are multifaceted research and teaching activities in the field of PE, which could be matched to 5 categories and 14 subcategories in total. Almost half of the publications originated from the USA, followed by Canada and the United Kingdom. Perhaps due to the fact that PE procedures and examination (e.g. OSCE) have been an established part of the Anglo-American medical training culture for longer than in Central Europe, but also notably because of the search strategy (non-English/non-German studies were excluded).

Interpretation of the results was limited due to study design, studies with low numbers of participants and the country-specific differences (in relation to curriculum, culture, heterogeneity of the used teaching methods).

Studies from Europe (excluding UK) accounted for less than 9% of publications.

Overall, non-randomized intervention studies represented the largest group (*n* = 91) followed by cross-sectional studies (*n* = 29).

Several authors advocate for more rigorous study design in the field of medical education also considering economic aspects. Furthermore, outcome- and process-based evaluations using a mixed-methods design are essential for understanding whether, why and how courses work [91–93]. The 117 articles were assigned to the following five categories: teaching methods, teaching quality, performance evaluation and examination formats, students’ views and patients’ and standardized patients views. With regard to the ‘teaching methods’ category (*n* = 53 articles), computer/internet based learning, simulator based learning or peer-assisted learning may have positive effects on learning PE and have the potential to increase the self-confidence of the students. Negative effects of the new approaches were not reported. It should be mentioned that in many studies results of former students were compared to results of students who underwent new teaching formats. The study results cannot confirm if the better outcome is directly related with the teaching method or with more study time in general. Core steps of musculoskeletal, neurological or general PE were assigned to the category learning quality (*n* = 33 articles) [67–70]. These studies may be helpful and informative for the development of learning goals plus help with the contents and scope of a step-wise PE. Successful teaching of PE-skills seems to be independent of the place where teaching of PE takes place (for example in hospitals or practices) [71]. This may support further involvement of primary care physicians in teaching PE. By doing so, students would have additional chances to become acquainted with the primary care sector. Nineteen articles were assigned to the category performance evaluation and examination formats. OSCE seems to be an appropriate examination format that is little influenced by external factors, such as the background of the examiner or the time of the day [76]. Mainly qualitative research methods (e. g. interviews) were used to study the perspectives of students. Studies concerning the willingness of participation in peer PE or DRE could be assigned to this category. Gender differences were found in the willingness of participation in peer PE. Females prefer being examined by a fellow student, furthermore non-white students and religious students were less willing than white students and non-religious students to examine and be examined by students of the opposite sex. Especially for examining intimate body regions students prefer same gender constellations [79]. In terms of the latter, simulation-based learning may be of benefit because students begin with DRE in a protected learning environment [46]. Patients highlighted the desire to have intimate examinations performed by trained or advanced students [90]. This could be supported by using simulation based learning approaches in first place for none or less experienced students.

The main reason patients participated in PE seems to be willingness to help (see category patients’ and standardized patients’ views). Patients and standardized patients should be adequately informed about the PE, its duration, the numbers of participants, and any assistants [88]. The learning success seems not to depend on the professional background of the PE teacher [76]. This may support further involvement of staff other than physicians (e.g. nurses, midwives) in the teaching of PE. Small group teaching requires extensive staff (and monetary) resources. Several studies compared the teaching of PE by physicians and non-physicians. In most of those studies, no significant differences in terms of the teaching quality could be observed [60–65]. Further utilization of advanced students could be of benefit in teaching PE skills [59].

### Answering the research question

The aim of this article was to scope the literature for teaching and research activities in the field of PE in undergraduate medical education in order to answer the research question. It can be postulated that the range of literature regarding different methods of teaching PE competences is very broad. More than 9 different teaching methods were found in the literature, especially the approaches computer−/internet-based courses, learning materials, concepts as well as the practical courses simulation-based learning, peer-assisted learning, bedside teaching, OSCE, anatomy and ultrasound-controlled courses were mentioned frequently. The *n* = 117 identified articles were matched into 5 categories, which represented the possible dimensions of assessment. The quality of physical examination teaching in undergraduate medical education is determined by qualification of the teachers, content quality and learning environment: Students who were taught by full-time faculty showed better performance in PE skills and gender aspects (same-gender versus mixed-gender partnering in practicing PE) were found to be of great relevance. Regarding study design, more than 50% of the identified studies were conceptualized as interventional, controlled studies. The most papers (*n* = 14) were published in the year 2009, with a slightly rising trend since 2000. The research institutions were typically located in Anglo-American countries.

### Limitations

Due to organizational reasons the literature search was restricted to the above mentioned search syntax and the Medline data base. Grey literature search or screening of the reference lists was not performed. Only English and German articles were included. Due to the very broad search strategy we had difficulties with the heterogeneity of the results in terms of categorization and interpretation. Unambiguous allocation to the categories was difficult and many articles could have been allocated to more than one category. We decided deliberately to assign every article only to one category.

## Conclusion

The present work shows a broad spectrum of research activity in the field of PE and a certain level of evidence for the effectiveness of individual teaching methods. It can be used to orientate and generate momentum for the development of medical education in the field of PE. For further improvement of the undergraduate medical teaching in PE further research is necessary regarding the content and extent of the PE. Coordination of the different medical specialists is certainly challenging as well as applying generalized results to country-specific educational structures, cultures and curricula. Nevertheless, the results of this scoping review can be used to inform others researchers, educators and medical professionals regarding the preceding research a round physical examination teaching and assessment. It will hopefully help in their own course development. Indeed, our own course development was significantly influenced by this content: In addition to simulation patients we decided to offer additional repetition courses using peer group teaching. Furthermore, we are planning to augment our course with e-learning modules in the near future.

## Abbreviations

DOCEE: Direct observation clinical encounter examination
DRE: Digital rectal examination
HTTE: Head-to-toe examination
IMCD: Internal medicine clerkship directors
OSCE: Objective structured clinical examination
OSVE: Objective structured video examination
PDCD: Physical diagnosis course directors
PE: Physical examination
PICOS-scheme: (Patients, Intervention; Control; Outcome; Study Design)
PPE: Peer physical examination
SP: Standardized Patients
